# Impacts of Methyl Farnesoate and 20-Hydroxyecdysone on Larval Mortality and Metamorphosis in the Kuruma Prawn *Marsupenaeus japonicus*

**DOI:** 10.3389/fendo.2020.00475

**Published:** 2020-07-28

**Authors:** Kenji Toyota, Fumihiro Yamane, Tsuyoshi Ohira

**Affiliations:** ^1^Department of Biological Sciences, Faculty of Science, Kanagawa University, Kanagawa, Japan; ^2^Department of Biological Science and Technology, Faculty of Industrial Science and Technology, Tokyo University of Science, Tokyo, Japan; ^3^Mie Prefectural Fish Farming Center, Shima, Japan

**Keywords:** crustacean, Penaeid, juvenile hormone, ecdysone, molting

## Abstract

Physiological functions of juvenile hormone (JH) and molting hormone have been demonstrated in insects. JH, molting hormone and their mimics (insect growth regulators, IGRs) show endocrine-disrupting effects not only on target pest insects but also on other arthropod species such as crustaceans. However, little is known about the endocrine-disrupting effects of IGRs on benthic crustaceans. In this study, laboratory experiments were conducted to investigate effects of representative innate JH in crustaceans (methyl farnesoate, MF) and molting hormone (20-hydroxyecdysone, 20E, active form of ecdysteroid) on larval stages of the kuruma prawn *Marsupenaeus japonicus*, which is a decapod crustacean living in warm seawater. Larval development of kuruma prawn progresses in the order of nauplius, zoea, mysis, and then post-larvae with molting and metamorphosis, but it is unknown whether both MF and 20E have crucial roles in metamorphosis and molting of this species. Treatments of either MF or 20E on shrimp larvae were attempted at each developmental stage and those effects were validated. In terms of EC_50_ values between mortality and metamorphosis, there were apparent differences in the transition from nauplius to zoea (MF: 7.67 and 0.12 μM; 20E: 3.84 and 0.06 μM in survival and metamorphic rates, respectively). In contrast, EC_50_ values in MF and 20E treatments showed high consistency in the transitions between zoea to mysis (EC_50_ values for survival; MF: 1.25 and 20E: 0.22 μM), and mysis to post-larvae (EC_50_ values for survival; MF: 0.65 and 20E: 0.46 μM). These data suggest that nauplius has strong resistance against exposure to MF and 20E. Moreover, both chemicals induced high mortality triggered by the disruption of molting associated with metamorphosis. To our knowledge, this is the first experimental evidence that investigates *in vivo* physiological functions of MF and 20E in the larval stages of kuruma prawn, shedding light on not only ecotoxicological impacts of IGRs released into nature, but also endocrine mechanisms underlying larval development with metamorphosis in benthic decapod crustaceans.

## Introduction

Recent advances in arthropod phylogeny have revealed that the Crustacean clade is not monophyletic, and can be divided into three extant clades (Ostracoda, Malacostraca, Branchiopoda). A current hypothesis supports that the clade of Hexapoda (insect species) is nested within the Crustacea, which forms a new clade known as Pancrustacea, although its details are still controversial (1, 2). This finding has provided impetus into the belief that the comparative analysis of crustaceans and insects is indispensable to understanding the evolutionary origin of a range of characteristics that are believed to be insect-specific. Indeed, both crustaceans and insects share various fundamental traits such as endocrine-driven developmental and reproductive processes, which are regulated primarily by juvenile hormone (JH) and molting hormone (ecdysteroids).

Methyl farnesoate (MF) is thought to be the equivalent of JH in crustaceans. Previous studies have demonstrated that MF may play a similar role to JH in insects, by regulating molting, sexual maturation, and reproduction in concert with ecdysteroids, the main active form being 20-hydroxyecdysone, or 20E (3–5). To date, the hormonal actions of MF and 20E are triggered by activation of the JH receptor (JHR) complex (methoprene-tolerant and steroid receptor coactivator) and ecdysone receptor complex (ecdysone receptor and ultraspiracle), respectively, which are the nuclear receptors responsible for transcriptional regulation of the aforementioned biological processes in crustaceans as well as insects (6–10). Based on those findings, endocrine-disrupting chemicals targeting the JHR and/or EcR have been designed and developed as insect growth regulators (IGRs) that disrupt metamorphosis and/or molting in pest arthropods resulting in the effective suppression of pest outbreaks (11, 12). However, due to highly conserved endocrine systems between insects and crustaceans, the environmental residues of these IGRs may also affect ecologically and economically important non-target species, such as aquatic crustaceans (e.g., prawns and crabs). Despite much earnest research to investigate the toxic effects of IGRs using tiny crustaceans such as water fleas, little is still known about endocrine-disrupting effects of IGRs on benthic crustaceans. This knowledge gap is largely due to a lack of established model crustacean species that can be applied for physiological and toxicological studies.

In Malacostracan crustaceans, the eyestalk neurosecretory system, which is referred to as the X-organ–sinus gland complex, plays a pivotal role in larval development associated with molting and metamorphosis. However, the endocrine mechanisms underlying larval development are still largely unknown. Generally, biosynthesis and secretion of ecdysteroids are negatively regulated by molt-inhibiting hormone, and those of MF are also suppressively controlled by mandibular organ-inhibiting hormone (MOIH) secreted from the X-organ–sinus gland complex in eyestalks, indicating that both endogenous ecdysteroids and MF titers increase when both eyestalks were ablated (13, 14). Based on this knowledge, numerous studies found that eyestalk ablation of larvae resulted in the formation of larval intermediates in the blue crab (*Callinectes sapidus*) and the American lobster (*Homarus americanus*), and took place in extra-larval stages causing a consequent delay in metamorphosis in the mud crab (*Rhithropanopeus harrisii*), shrimps (*Palaemon macrodactylus, Palaemonetes varians*), and in the swimming crab (*Portunus trituberculatus*) (15) reviewed in (4). However, the mechanisms by which these hormones exert their effects remain poorly understood.

Kuruma prawn, *Marsupenaeus japonicus*, is a member of the family Penaeidae (Class Malacostraca, Order Decapoda), and is widely distributed from Japan and Southeast Asia to Western Pacific Oceans (16). Due to the economic importance of this species, annual catches have declined sharply since the 1990s (17). To overcome this situation, research into seed production of kuruma prawn, which was reared from eggs to juveniles that were then released into sea water to maintain natural populations, was conducted (18). Based on a long history of seed production of kuruma prawn, its larval developmental process is well-described. Nauplius larvae hatch about 13–14 h after ovulation at 27–29°C, and repeat molting six times (stages I–VI) within 36 h, resulting in metamorphosis to the zoea stage. Likewise, the zoea molts three times (stages I–III) within 4 days and metamorphoses into the mysis stage. Finally, the mysis molts three times (stages I–III) within 3 days and metamorphoses into the post-larval stage (4). Although 20E-driven ecdysteroid signaling pathways are known to regulate molting in crustaceans, less is known about the regulation of larval development and metamorphosis by MF and 20E. In this study, we treated kuruma prawn larvae at each developmental stage with either MF or 20E and validated toxic effects such as rates of mortality and metamorphosis.

## Materials and Methods

### Animals

Sexually matured female kuruma prawns (*M. japonicus*) were purchased from a local fishery shop at a fishing port of Isshiki in Nishio City, Aichi Prefecture, Japan, in April 2018. All prawns were transferred to the Mie Prefectural Fish Farming Center (Mie, Japan) and all experiments were conducted there. Prawns were maintained in a tank with natural seawater at 24°C under natural daylight and fed daily with polychaeta worms as raw bait for 1 day prior to treatment of eyestalk ablation that stimulates ovarian maturation and then spawning within a few days. Newly hatched nauplius larvae (*ca*. 10,000 individuals), which were obtained from seven females, were transferred to a 100 L black tank with natural seawater at constant 24°C under natural daylight. The culture feed series of kuruma prawn larvae was as follows: from egg to zoea stage I (3 days after beginning) was the commercial diatom *Chaetoceros gracilis* (Pacific Trading Co., Ltd., Fukuoka, Japan); from zoea stage 2 to mysis stage I was both commercial diatom and a prawn diet (Vitalprawn: Higashimaru Co., Ltd., Kagoshima, Japan); thereafter, both the commercial prawn diet and nauplius *Artemia* larvae were added until prawns grew to the post-larval stage.

### Preparation and Treatment of Chemicals

A stock solution of 10 mg/mL MF (Echelon Bioscience, Salt Lake City, UT, USA) was dissolved in 100% ethanol (EtOH, Wako Pure Chemical Industries Ltd., Osaka, Japan) and kept at −20°C until use. Based on this stock solution, a dilution series was prepared, as follows: 5.0, 2.5, 1.25, 0.625, and 0.3125 mg/mL. These stock solutions were directly added to 500 mL of natural seawater containing 10 individuals each. Likewise, a stock of 100 mg/mL 20E (Sigma-Aldrich, St. Louis, MO, USA) was dissolved in 100% EtOH and kept at −20°C until use. A second dilution series was prepared as follows: 50, 25, 12.5, 6.25, and 3.125 mg/mL. These stock solutions were directly added to 500 mL of natural seawater containing 10 individuals each. Beakers were prepared in triplicate for each condition.

The final concentrations of MF for nauplius stage IV were 32.0, 16.0, 8.0, 4.0, 2.0, 1.0, 0.5, 0.25, 0.125 μM. Then, based on result of nauplius experiment, the range of concentrations of experiment of both zoea stage III and mysis stage III was decided as 2.0, 1.0, 0.5, and 0.25 μM. Similarly, the final concentrations of 20E for nauplius stage IV were 16.0, 8.0, 4.0, 4.0, 2.0, 1.0, 0.5, 0.25, 0.125, and 0.0625 μM, and for both zoea stage III and mysis stage III, they were 2.0, 1.0, 0.5, and 0.25 μM.

After 24 h, starting from hatching, most prawns grow to nauplius stage IV. Those were exposed to MF or 20E, and mortality, molting, and metamorphosis (from nauplius stage VI to zoea stage I) were recorded for 72 h. The half maximal effective concentrations (EC_50_) and 95% confidence interval (CI) of mortality and metamorphosis rates of tested chemicals were calculated using R software (19). All data is available upon request.

## Results

### Effects of MF and 20E on the Nauplius Stage

**Figure 1:**
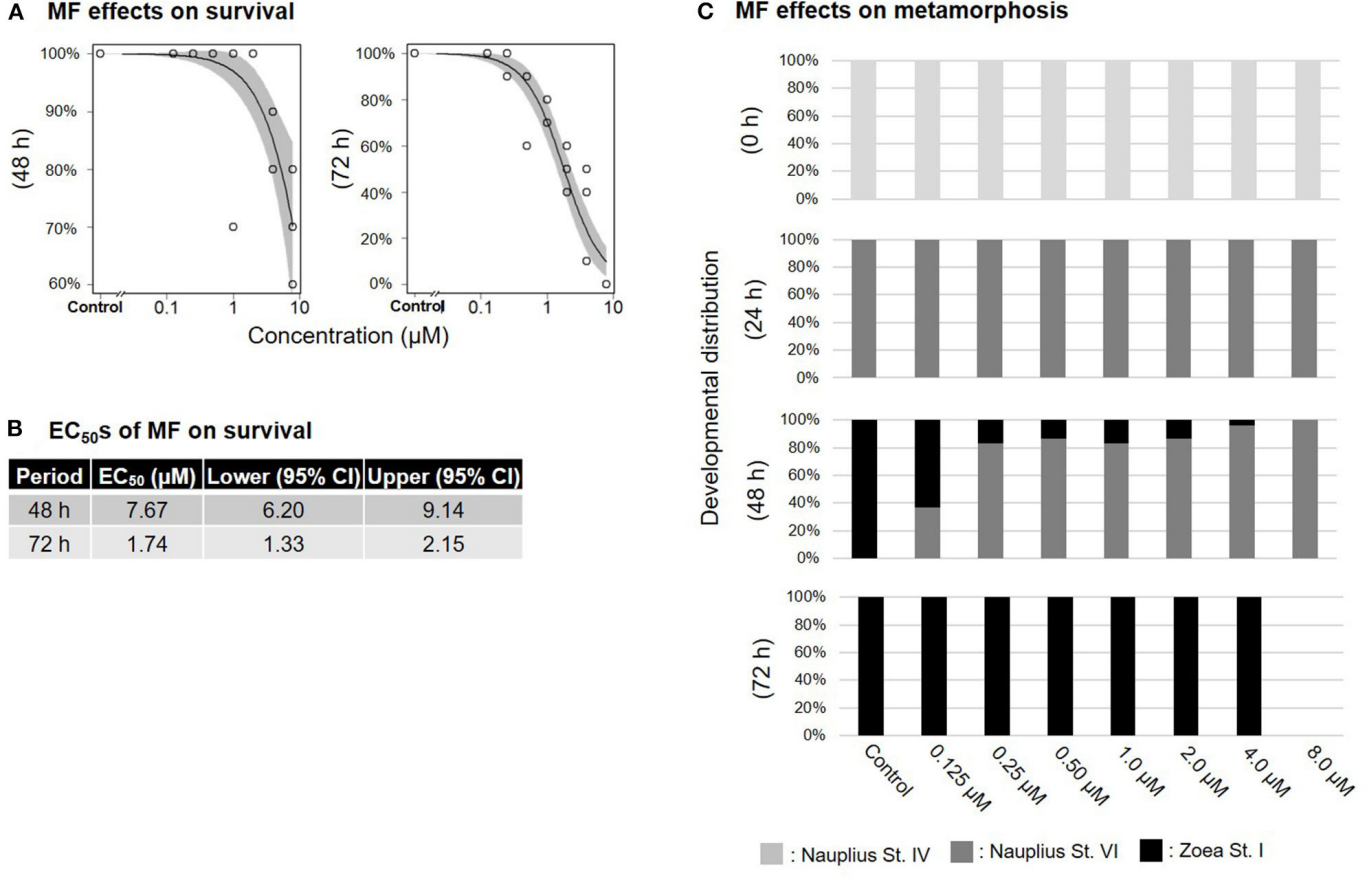
Effects of MF treatment on nauplius stage IV. Regression curves of survival rate at 48 and 72 h **(A)**. Gray shade indicates 95% confidence interval. Each EC_50_ value of survival rate at 48 and 72 h **(B)**. Metamorphosis rates after exposure to MF at 0, 24, 48, and 72 h **(C)**.

**Figure 2:**
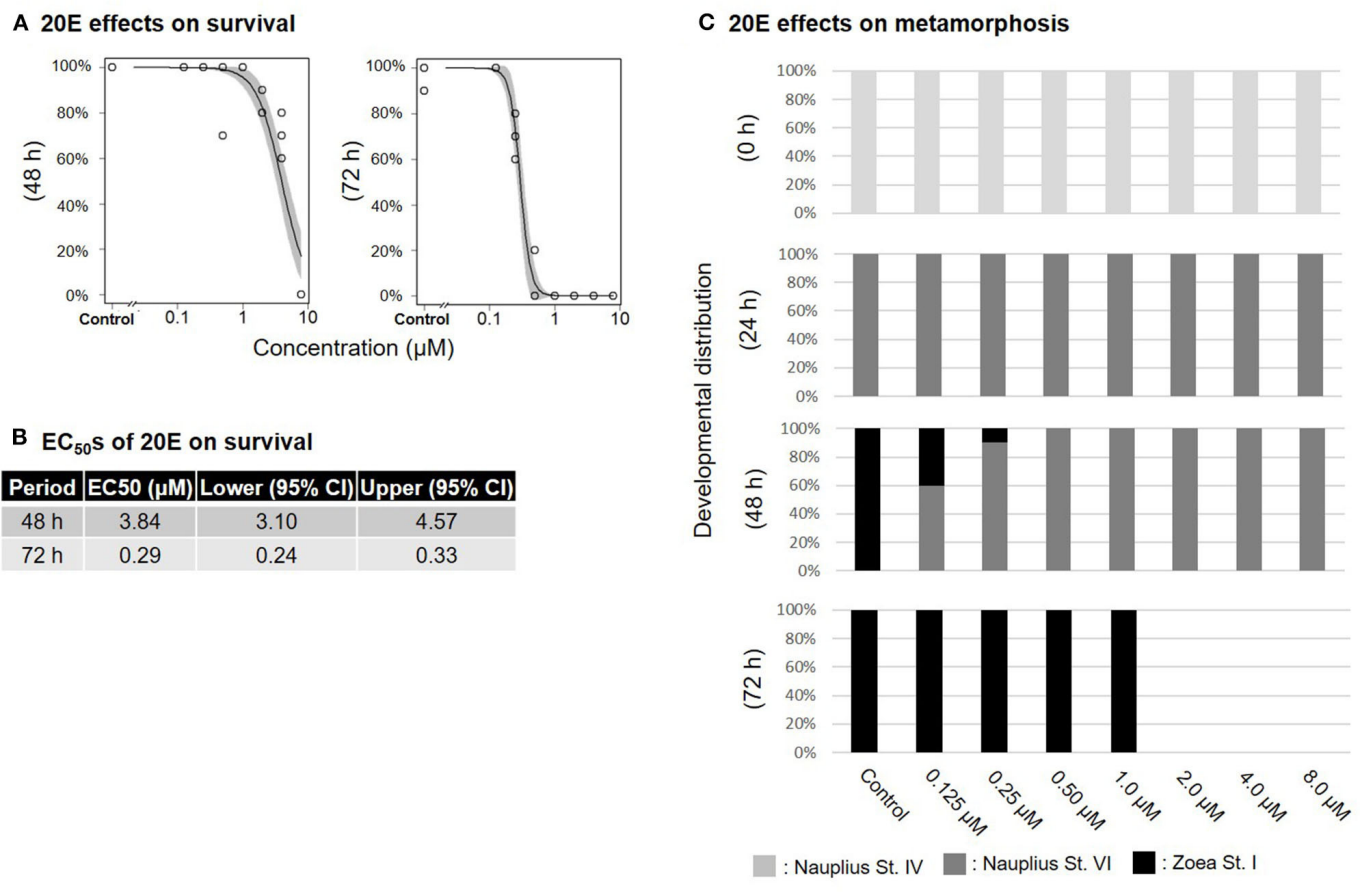
Effects of 20E treatment on nauplius stage IV. Regression curves of survival rate at 48 and 72 h **(A)**. Gray shade indicates 95% confidence interval. Each EC_50_ value of survival rate at 48 and 72 h **(B)**. Metamorphosis rates after exposure to 20E at 0, 24, 48, and 72 h **(C)**.

Treatment of nauplius stage IV prawns with either MF or 20E caused a dose-dependent decline in survival rates, and EC_50_ values at 72 h were apparently lower (MF: 1.74 μM; 20E: 0.29 μM) than at 48 h (MF: 7.67 μM; 20E: 3.84 μM) (Figures 1A,B, 2A,B). In the solvent control group, metamorphic transitions from nauplius stage VI to zoea stage I occurred within 48 h, but metamorphosis was delayed in a dose-dependent manner in both MF and 20E treatment groups (Figures 1C, 2C). The EC_50_ values of metamorphosis for MF and 20E were 0.12 (95% CI: 0.05–0.20) and 0.06 (95% CI: 0.04–0.07) μM, respectively. No metamorphosis took place after exposure to 8.0 μM MF and >2.0 μM 20E. All individuals that died during this experiment were exclusively nauplius at stage VI. Moreover, transitions from nauplius stage IV to stage VI occurred at 24 h after initial exposure in all treatment groups (Figures 1C, 2C). The results show that 20E was more toxic than MF to the nauplius stage in terms of the rate of mortality and metamorphosis.

### Effects of MF and 20E on the Zoea Stage

**Figure 3:**
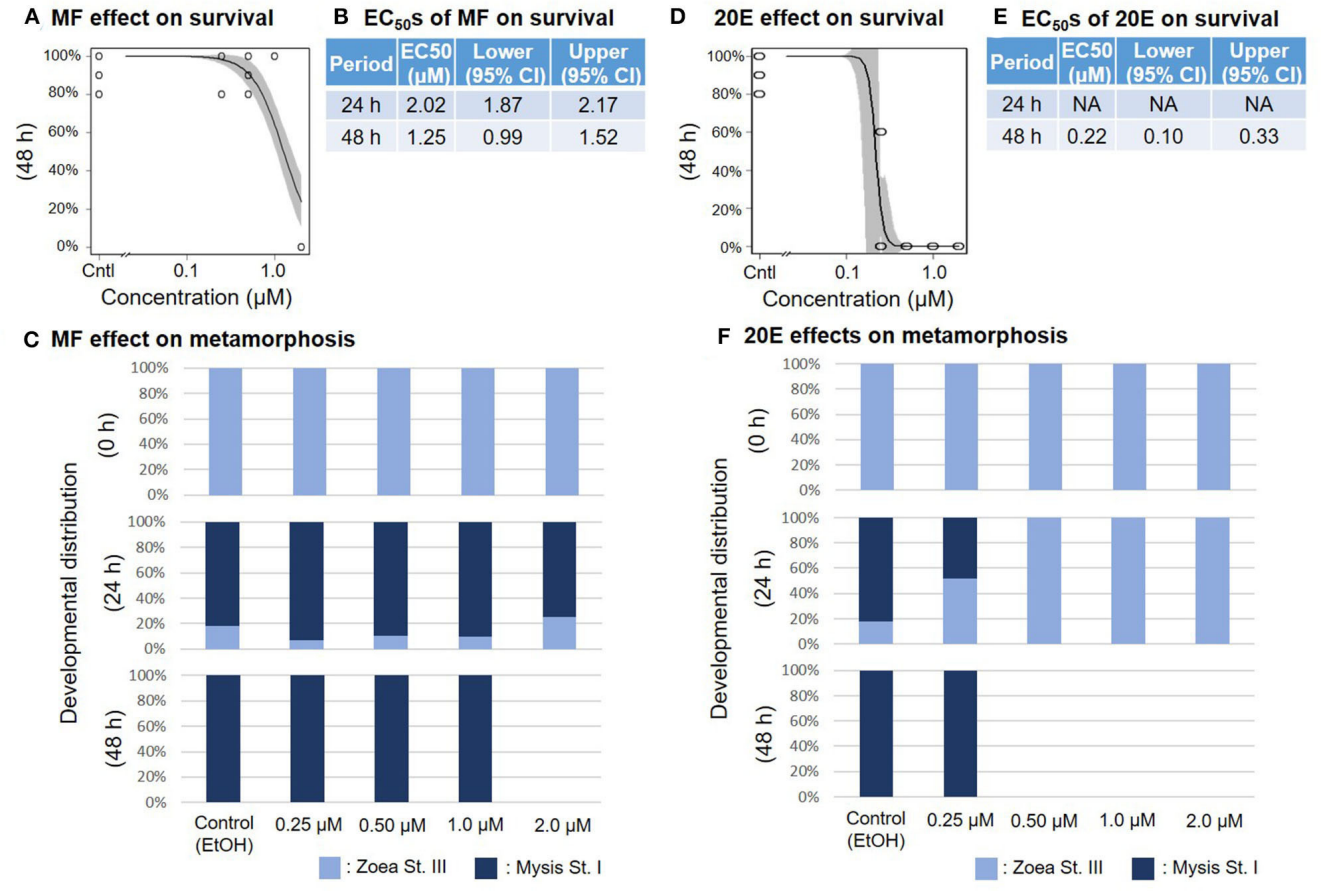
Effects of MF and 20E treatments on zoea stage III. Regression curves of survival rate at 48 and 72 h in response to MF and 20E treatments **(A,D)**. Gray shades indicate 95% confidence intervals. EC_50_ values of MF and 20E treatments **(B,E)**. Metamorphosis rates after exposure to MF and 20E at 0, 24, and 48 h **(C,F)**.

When the zoea larvae at stage III were exposed to a concentration series of MF or 20E, survival ratios decreased sharply in a dose-dependent manner at 48 h (Figures 3A,D). The EC_50_ values of survivability of zoea larvae for MF and 20E were 1.25 (95% CI: 0.99–1.52) and 0.22 (95% CI: 0.10–0.33) μM, respectively (Figures 3B,E). Although the metamorphic transition from zoea to mysis occurs normally within 48 h, no metamorphic transition occurred in the 20E treatment at more than 0.5 μM, unlike in the MF treatment groups (Figures 3C,F), and all individuals that died were zoea at stage III, except for the 2.0 μM MF treatment group.

### Effects of MF and 20E on the Mysis Stage

**Figure 4:**
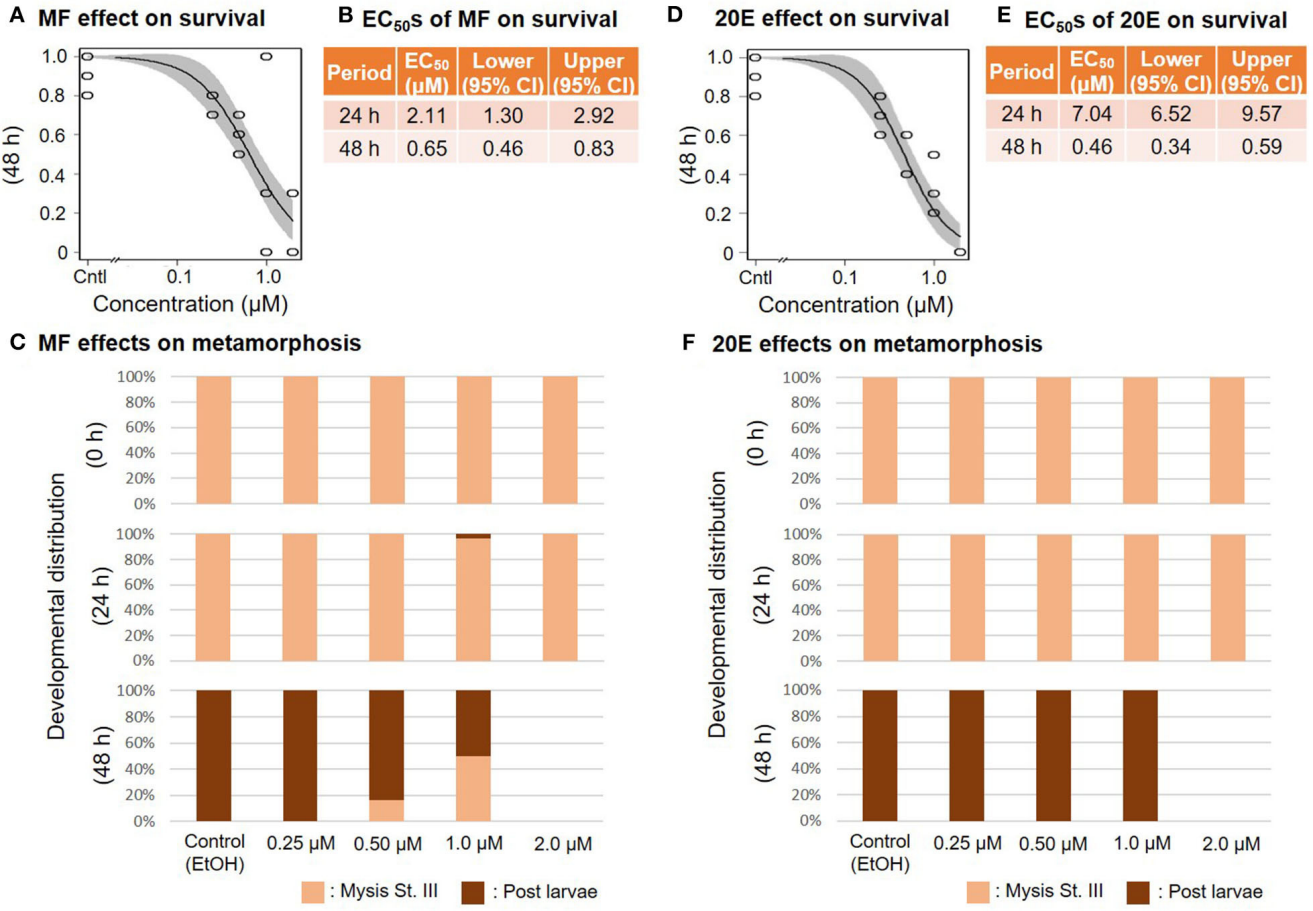
Effects of MF and 20E treatments on mysis stage III. Regression curves of survival rate at 48 and 72 h in response to MF and 20E treatments **(A,D)**. Gray shades indicate 95% confidence intervals. EC_50_ values of MF and 20E treatments **(B,E)**. Metamorphosis rates after exposure to MF and 20E at 0, 24, and 48 h **(C,F)**.

When mysis larvae at stage III were exposed to a concentration series of MF or 20E, survival ratios decreased dose-dependently at 48 and at 24 h, respectively (Figure 4). The EC_50_ values of 48 h survivability of mysis lavae for MF and 20E were 0.65 (95% CI: 0.46–0.83) and 0.46 (95% CI: 0.34–0.59) μM, respectively (Figures 4B,E). Even though the metamorphic transition from mysis to post-larvae occurs normally within 48 h, there was a dose-dependent decrease in metamorphic rate in response to MF treatments. In contrast, post-larvae were found in the 20E concentration series. No prawns survived exposure to 2.0 μM of either MF or 20E (Figures 4C,F). All individuals that died during this experiment were stage III mysis.

## Discussion

**Figure 5:**
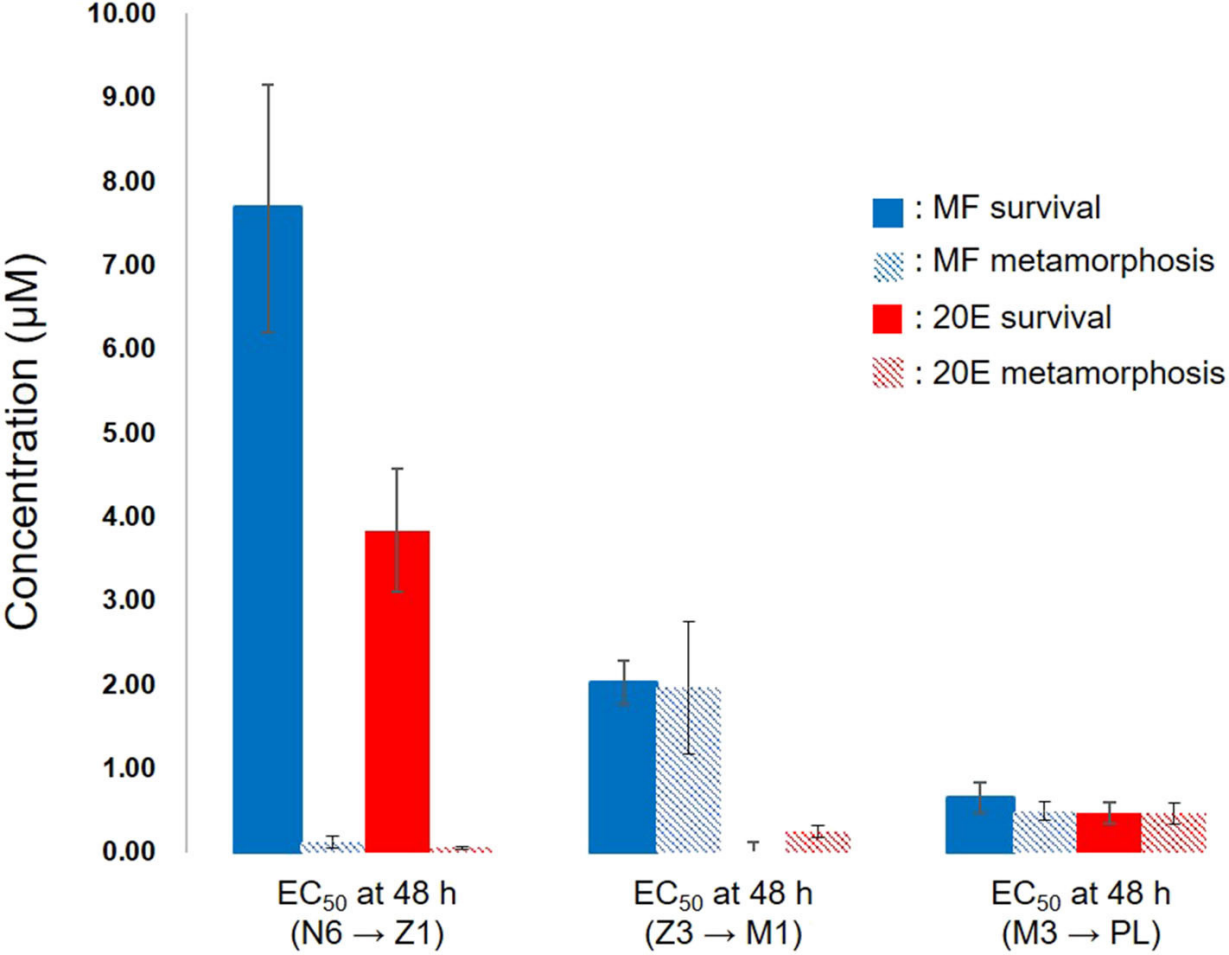
Relationships between EC_50_ of survivability and metamorphosis at 48 h after exposure of MF and 20E. N6, nauplius stage VI; Z1, zoea stage I; Z3, zoea stage III; M1, mysis stage I; M3, mysis stage III; PL, post-larvae. Error bars indicate 95% confidence intervals. NA indicates that data is not available.

This study demonstrated that exogenous treatment of either MF or 20E to larval stages of kuruma prawn caused the retardation of molting associated with metamorphosis and a decline in survivability of larvae in a dose-dependent manner. Interestingly, EC_50_ values at 48 h after the exposure to these compounds indicated that 20E more effectively decreased survivability and delayed metamorphosis than MF (Figure 5). At the nauplius stage, there were the huge gaps in EC_50_ values between survival and metamorphosis in both MF and 20E treatments. However, each EC_50_ value between MF and 20E was very consistent when transitions occurred from zoea to mysis, and from mysis to post-larva (Figure 5). These findings suggest that nauplius larvae have higher tolerance against the lethal effect of MF and 20E than other stages (e.g., zoea and mysis), and that there are different endocrine cassettes regulating transition from nauplius to zoea and later metamorphosis (zoea to mysis, and mysis to post larvae stages). In terms of the effects of MF on larval development and metamorphosis, some studies have reported similar results, such as the administration of MF, which delayed larval development and metamorphosis in freshwater prawn (*Macrobrachium rosenbergii*) (20, 21), or MF treatment, which induced precocious metamorphosis in the barnacle (*Balanus amphitrite*) (22, 23). Likewise, the precise regulation of the endogenous level of 20E plays a key role in the success of molting. Supporting evidence from various crustaceans consistently suggests that a pulse of the endogenous 20E titer is required for a complete molting cycle (12, 24).

Our data showed that either exogenous MF or 20E treatment to kuruma prawn larvae decreased the survival rate, and retarded larval development and metamorphosis. The EC_50_ values that we observed were similar to those in previous studies using the freshwater tiny crustacean (*Daphnia magna*), which is a well-known environmental indicator. For example, mortality in response to 20E treatment occurred with 5.119 μM (25), and males were induced by treatment with 0.278 μM MF (26). Moreover, using chemically-synthesized insecticides with MF- or 20E-like bioactivity, many studies investigated their endocrine-disrupting effects in various crustaceans. In the mud crab (*R. harrisii*), treatment with 0.159 μM fenoxycarb reduced survival and extended the duration of larval metamorphosis from zoea to megalopa (27). The LC_50_ (50% lethal concentration) values at 24 h for fenoxycarb and methoprene, two JH-mimicking chemicals, were 4.74 and 6.31 μM, respectively (at 48 h, values were 3.52 and 4.48 μM), in the cherry shrimp (*Neocaridina davidi*) (28). Natural concentrations of methoprene in freshwater have typically ranged from 3.0 to 30.0 nM (29, 30), suggesting that IGRs with JH-activity might have toxic effects on crustacean development in wild populations. Similarly, treatment with 3.37–33.7 μM of RH 5849 (1,2-dibenzoyl,l-t-butyhly drazine), which has IGR-bearing ecdysteroid activity, accelerated larval molting in the crab (*R. harrisii*) and enhanced attachment and metamorphosis in the barnacle (*B. amphitrite*) (31), while exogenous treatment with 0.5 μM 20E inhibited molting and ovulation in the water flea (*D. magna*) (32). Taken together with findings in other crustaceans, the present study demonstrates that larval development in the kuruma prawn may be highly sensitive to MF- and 20E-like chemicals. In addition to short-term assay, the long-term (chronic) assay will be more clearly demonstrated that impacts of those chemical exposure on larval development of kuruma prawn in the ecological point of view.

Recent advances in omics approaches enable the depiction of the expression pattern of many genes and to estimate the regulatory interactions involved in metamorphosis in the prawn (*M. rosenbergii*) (33), the shrimp (*Neocaridina denticulata*) (34), and the spiny lobster (*Sagmariasus verreauxi*) (35, 36). Although the aforementioned transcriptome studies provided various new insights, the number of unannotated genes has hampered the completion of more comprehensive analysis due to the lack of publicly available genomes. To address this resource problem, the complete genome of the Pacific white shrimp (*Litopenaeus vannamei*) was decoded, providing a new hypothesis of the regulatory mechanisms underlying adult molting via sterol regulatory elements (SRE)-binding protein and opsin (37). In addition to next generation sequencing, mass spectrometry technology (e.g., LC- and GC-MS) has allowed the metabolite profiling (38), and quantification of endogenous juvenoid in hemolymph of freshwater prawn *M. rosenbergii* (39) and ecdysteroid titers in the extracts of tiny crustaceans (40, 41), enabling the monitoring of the pulse (rise and decline) of those hormones during metamorphosis. It will be necessary to elucidate the fluctuating dynamics of MF and 20E titers during larval development in kuruma prawn. Some advanced studies found that this can be successfully achieved by integrating the data acquired from *in vivo* pharmacological assays and omics approaches (28, 42, 43), suggesting that this approach will be applied for comprehensively understanding the mechanisms of diversified crustacean metamorphosis. Additionally, treatments of those inhibitors/antagonists will be useful for understanding their physiological function. Indeed, the fenarimol, which is an inhibitor of ecdysteroid synthesis, could be applied in the molting research, because it has been used in *Daphnia* (40). Although less is known about the inhibitor/antagonist of JH, some potential JH antagonists have been identified using the yeast two-hybrid system transformed with the mosquito JH receptor as a reporter system (44).

In conclusion, we conducted laboratory experiments to investigate the toxic effects of MF and 20E using larval stages of the kuruma prawn. We demonstrated that both MF and 20E induced high mortality caused by disruption of molting-associated metamorphosis, although the nauplius showed strong resistance to MF and 20E. This is the first experimental evidence of the *in vivo* physiological functions of MF and 20E in the larval stages of kuruma prawn, shedding light on not only the ecotoxicological impacts of IGRs released into nature, but also on the endocrine mechanisms underlying larval development with metamorphosis in benthic decapod crustaceans.

## Data Availability Statement

The raw data supporting the conclusions of this article will be made available by the authors, without undue reservation.

## Author Contributions

KT and TO designed all experiments in this study. KT and FY conducted all experiments and obtained all raw data. KT conducted all data analyses and prepared the first draft of the paper. All authors contributed to the article and approved the submitted version.

## Conflict of Interest

The authors declare that the research was conducted in the absence of any commercial or financial relationships that could be construed as a potential conflict of interest.
